# Supplementary material for: SAW Resonators and Filters Based on Sc0.43Al0.57N on Single Crystal and Polycrystalline Diamond
Source: Micromachines (Basel). 2022 Jun 30;13(7):1061. doi: 10.3390/mi13071061 (PMC9316532; doi:10.3390/mi13071061)
Supplement: Supplementary file 1 [file micromachines-13-01061-s001.zip › micromachines-1782609-supplementary.pdf]

Supplementary information

SAW Resonators and Filters Based on $Sc_{0.43}Al_{0.57}N$ on Single Crystal and Polycrystalline Diamond

SEM:

SEM inspection of the grounded reflector and IDT of the SAW filter.

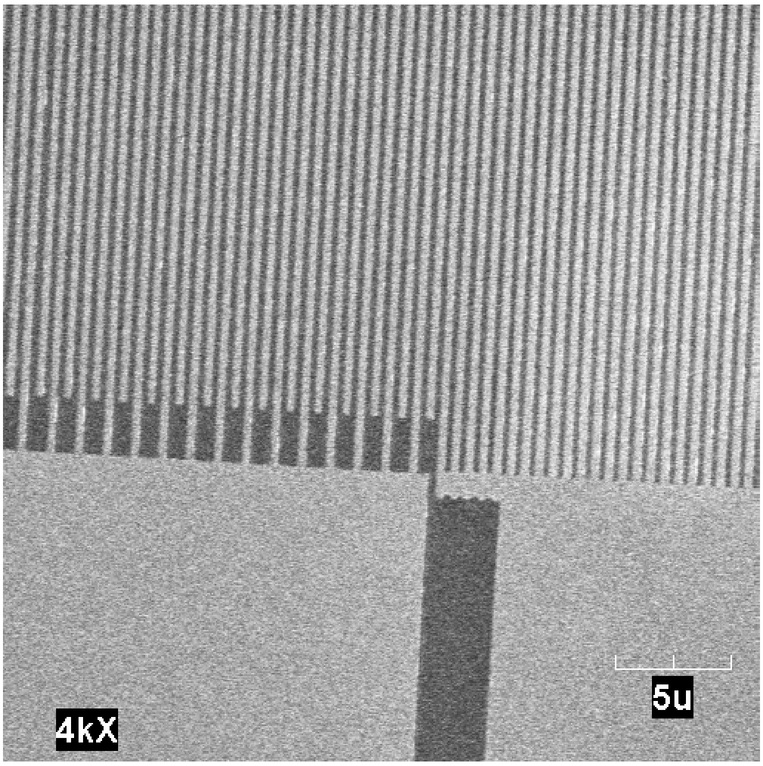

Figure S1. SEM inspection of the SAW filter IDT and ground reflector

RBS*:*

Rutherford Backscattering Spectrometry (RBS) (Figure S2)was carried out with a 3080 keV He++ beam. Measuring at this energy enhances the oxygen signal from the ScAlN layer because of the resonance in the He-O cross section at 3.05 MeV. The angle between the ion beam and the sample surface normal was 7° and the angle between the surface normal and the detector was 3°, so that the backscattering angle was 170°. Concentrations and thicknesses are obtained by comparing the experimental data with simulations done with the SIMNRA program (https://mam.home.ipp.mpg.de/index.html).

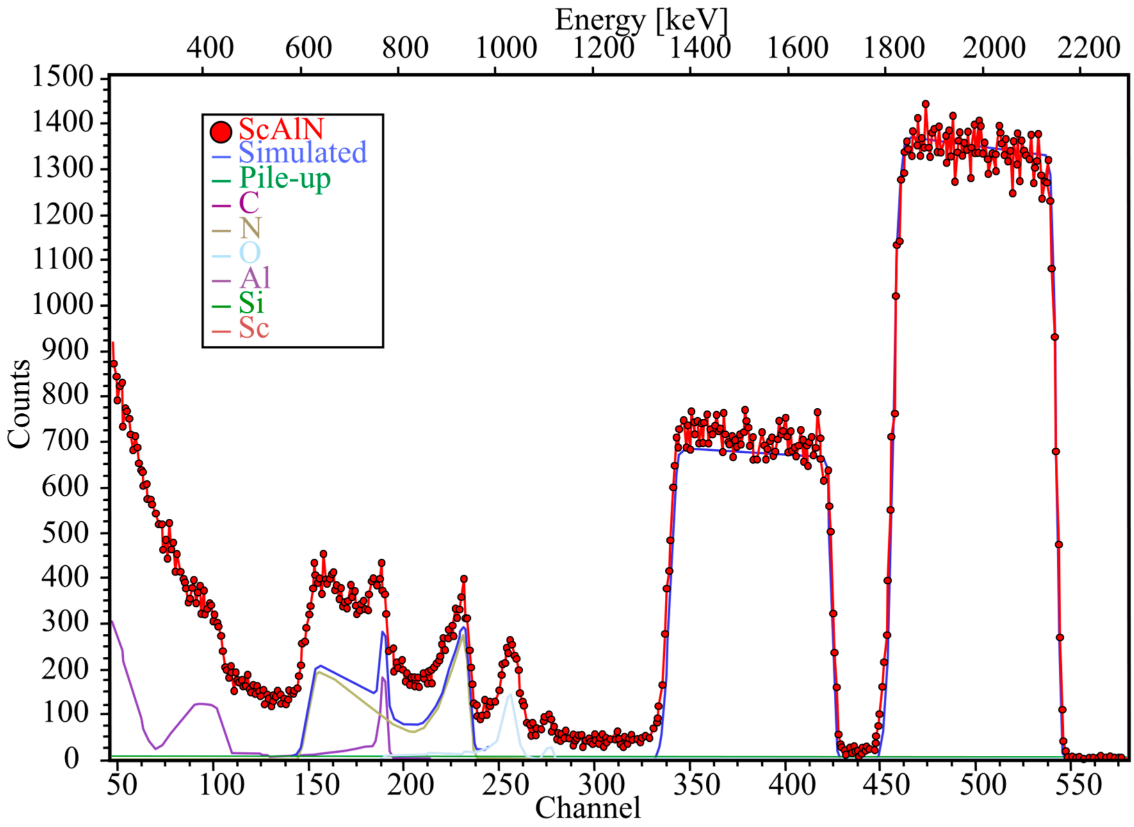

Figure S2. RBS spectra of a $Sc_{0.43}Al_{0.57}N$ thin film
